# Understanding the Mechanisms of Recovery and/or Compensation following Injury

**DOI:** 10.1155/2017/7125057

**Published:** 2017-04-20

**Authors:** Michael J. Hylin, Abigail L. Kerr, Ryan Holden

**Affiliations:** ^1^Neurotrauma and Rehabilitation Laboratory, Department of Psychology, Southern Illinois University, Carbondale, IL, USA; ^2^Department of Psychology, Illinois Wesleyan University, Bloomington, IL, USA

## Abstract

Injury due to stroke and traumatic brain injury result in significant long-term effects upon behavioral functioning. One central question to rehabilitation research is whether the nature of behavioral improvement observed is due to recovery or the development of compensatory mechanisms. The nature of functional improvement can be viewed from the perspective of behavioral changes or changes in neuroanatomical plasticity that follows. Research suggests that these changes correspond to each other in a bidirectional manner. Mechanisms surrounding phenomena like neural plasticity may offer an opportunity to explain how variables such as experience can impact improvement and influence the definition of recovery. What is more, the intensity of the rehabilitative experiences may influence the ability to recover function and support functional improvement of behavior. All of this impacts how researchers, clinicians, and medical professionals utilize rehabilitation.

## 1. Recovery or Compensation

One important question in the study of adult brain injury and repair is whether behavioral improvement reflects true behavioral recovery or whether the behavioral changes are simply due to the use of compensatory strategies in reaction to a disrupted nervous system. Part of the difficulty with recovery, from a clinical perspective, is in how it is defined. To a clinician, recovery may be generally defined in terms of improved behavior following an injury. Good recovery is defined by the Glasgow Outcome Scale as a “resumption of normal activities even though there may be minor neurological and psychophysical deficits” [1, 2]. Therefore, from the clinical perspective, an individual is “recovered” if they are able to show some improvement in functioning independently [3, 4]. Still, defining recovery as simply returning to normal life is a fairly limited concept in that it does not take into account whether the individual is simply compensating for their lost behaviors. Foroud and Whishaw [5] analyzed the kinematic profiles of two stroke patients during a reaching task and found that while the patients were able to perform the task, they demonstrated kinematic abnormalities during reaching. Though in only two patients, this evidence of compensatory limb use suggests a qualitatively different functional outcome than true behavioral recovery. These qualitative differences are likely reflected in central nervous system structure and function, the effects of which may have long-term impacts on behavioral outcome.

Typically, the focus of rehabilitative treatments following stroke or traumatic brain injury is often upon encouraging the development of compensatory strategies in order to resume independent daily living [6–8]. For instance, individuals that are hemiplegic have difficulty using their impaired limbs and rehabilitative therapy may encourage the use of their unimpaired side. Patients may also use compensatory strategies to cope with cognitive impairments. For example, if an individual is impaired in their ability to remember, a clinician may encourage the use of a journal or diary to help them remember daily events [9–12]. However, in both of these examples, even though the individual may be equipped to return to daily independent living, their impairments are still present and may interfere with long-term functional outcome instead of focusing on encouraging the lost behavior to return; the focus is upon compensating for their loss. Essentially, the goal of rehabilitation is often focused on independence as opposed to the impairment, which may lead to improved behavioral function but prevent true behavioral recovery.

Up to this point, it could be argued that the scenarios discussed illustrate how individuals are able to compensate for the lost behavior. In fact, it is important to point out that sometimes even when an individual appears to have “recovered” and is able to function independently, they may continue to show “silent” cognitive and motor deficits in areas such as emotion, attention, or fine motor control [13–19]. These deficits are important to take into consideration in discussing whether an individual has recovered as they may otherwise go ignored. Usually, depending upon the severity of the injury, deficits will abate over time, although some residual deficits may remain [20–22]. In fact, some authors have questioned whether it is possible to observe true recovery [23–25]. If we hope to improve the efficacy of rehabilitative strategies following brain injury, it is important to distinguish between true recovery and compensation and to understand the consequences of each for long-term neural and behavioral function. This review focuses on the anatomical and behavioral mechanisms surrounding the distinction between recovery and compensation in the adult with the aim of using this distinction to guide strategies for successful rehabilitation. As the majority of human strokes are ischemic in nature, we focus specifically on ischemic insult in the adult (human, nonhuman primate, and rodent models) [26]. Traumatic brain injuries (TBI) have a fairly distinct pathological response that is separate from stroke [27, 28]. While within TBI characterization, focal and diffuse injuries have distinct pathologies; most TBIs have elements of both types of injury and are heterogeneous in nature making the distinction difficult from the perspective of recovery and treatment [29–31]. In addition, traumatic axonal injury has been shown to lead to selective atrophy in a regional manner similar to what is observed following focal injury [32]. This review considers how the cognitive reserve applies to functional improvement following injury and how the intensity of rehabilitation plays a role in recovery and compensation.

## 2. Neuroanatomical Effects of Damage upon Recovery/Compensation

In understanding how compensatory strategies are developed, one must consider the anatomical and cellular events that are associated with injury. Following unilateral brain injury, there is a sequence of events that have devastating effects upon the primary site of damage. Regardless of how the damage occurs (e.g., ischemic stroke or TBI), this usually begins with the interruption of the blood supply to the brain, which causes cells to die through over excitation due to excess glutamate release [33–38]. Following the initial damage, tissues become swollen and inflamed, compromising the integrity of areas that are distant from the primary site of damage [37, 39–42]. This swelling and inflammation may cause deficits that will abate over time [43, 44]. There is also a decrease in the metabolic activity in the ipsilesional hemisphere suggesting that the damage can impact areas distal to the injury, a phenomenon known as diaschisis [45–47].

While injury results in devastating events, it also induces a cascade of growth-related events that enable remaining neurons near and distant to the lesion locus to survive, repair, and form new connections [48–53]. Some researchers have even proposed that some of the events that occur following damage are similar to what is observed during normal development, possibly suggesting that the brain has an intrinsic ability to react to change [54–59]. Changes in perilesion cortex (i.e., the area of vulnerable but surviving tissue immediately around the lesion core), including cortical reorganization, neurogenesis, axonal sprouting, dendritic plasticity, and angiogenesis, have been linked to spontaneous recovery of behavioral deficits following the resolution of diaschisis as described above [60–65]. Within the perilesion area, there is also an increase in the expression of growth-promoting genes which begins to be seen shortly after injury [66, 67]. This altered environment is arguably becoming growth permissive, as increased axonal sprouting will occur in this region [53, 68]. Increased levels of GAP-43, a marker for the presence of growth cones, have been observed shortly after injury [69, 70]. Carmichael and Chesselet [71] found that the increased axonal sprouting correlates with an altered physiological response in the perilesion area. This increase in synchronous activity is followed by activity in other cortical regions associated with the damaged area including the contralesional homotopic cortex. Further, it is likely that the axonal sprouting and altered activity in this region is what underlies reorganization of the remaining cortical representations in the perilesion area [42, 68, 71–75].

The perilesion area is not the only location that is altered in response to injury. Frost et al. [21] found that following lesions of the hand area of the primary motor cortex in primates, there is an expansion of the hand area of the ventral premotor cortex (PMv) in the same hemisphere [76]. This increased expansion is associated with altered axonal sprouting from the PMv [72, 77]. Both of these changes are correlated with improved motor behavior. Following injury, the contralesional hemisphere also exhibits an increase in dendritic growth [78–80] as well as increased sprouting of corticostriatal axons [78, 81]. Further, reorganization of contralesional hemisphere usually corresponds to increased synapse number [82, 83]. Some authors have suggested that this growth is the result of a compensatory behavioral response (e.g., [80, 84]).

Compensatory sprouting has also been looked at in models of TBI, as diffuse axonal injury is frequently observed following injury. The temporal lobes and hippocampus are one of the most vulnerable areas after a TBI [85, 86]. Injury results in robust neural plastic changes in the hippocampus, which include increased synaptogenesis, increased expression plasticity-related proteins such as extracellular signal-regulated kinase, and altered expression of genes associated with structural changes [87–93]. However, it remains to be determined if these changes reflect a positive impact following injury and if methods inducing recovery could employ them to support improved outcome. Utilizing a model of combined TBI with entorhinal cortex deafferentation, Philips et al. (1994) observed cognitive deficits (impairments in the water maze) that were associated with aberrant neural sprouting and synapse formation. Damage to the entorhinal cortex has been shown to produce reactive synaptogenesis and collateral sprouting and result in the formation of novel synapses in the dentate gyrus [94–96]. In patients following TBI, compensatory neural tracts extending from the contralesional fornix have been observed and may underlie recovery of memory impairments [97].

It appears that the brain is able to undergo neuroanatomical changes that lead to the reorganization of remaining tissue following damage. However, this reorganization has behavioral consequences that need to be considered when determining whether recovery or compensation has occurred.

## 3. Behavioral Effects of Recovery/Compensation

Instead of regarding recovery as a “general” improvement in behavioral functioning, researchers have a more varied and sometimes less well-defined criterion. One view of behavioral recovery is whether an end point has been achieved that is similar to the preoperative performance of the animal or to the performance of a nonlesioned animal [22, 98–100]. For instance, if an animal is able to learn to navigate a maze successfully, even if it takes more trials to learn than an intact control, one could argue that the animal has demonstrated functional recovery [101–104]. A similar result is seen with animals that have received motor cortex lesions. Initially animals are impaired in their ability to reach for food, but they will eventually be able to successfully improve their reaching behavior following weeks of testing [105]. However, it is important to point out that while they are able to become more successful over time they are still impaired relative to even their own preoperative baseline [24]. Depending upon the task, animals usually show some improvement in behavior over time, possibly suggesting that some form of “recovery” may be possible [106, 107]. It should be noted that with focused training of the impaired limb, animals often reach preoperative performance levels [108–111], suggesting that behavior can interact with naturally occurring plastic changes following stroke to drive functional outcome.

Often, behavioral changes are associated with the presence of neuroanatomical changes in areas functionally related to the damage. Following a unilateral lesion of the sensorimotor cortex, there is an increased reliance upon the unimpaired limb for movement and postural support which coincides with increased dendritic growth in the contralesional hemisphere [46, 47, 79]. The increased growth peaks at three weeks following injury, and pruning of this over growth begins to occur over the next few weeks. The overreliance on the unimpaired limb will also begin to decrease shortly after the dendritic pruning begins, possibly suggesting that the behavior of the animal over time following injury influences the ability for plasticity to occur [103, 112]. Jones and Schallert [80] tested this by restricting the movement of the unimpaired limb and forcing the animal to use their impaired limb. By restricting the unimpaired limb, the increased growth was blocked suggesting that the plasticity that occurs due to injury interacts with the behavioral deficit [80, 113]. Similar results have been found in rodent models of TBI, although the mechanistic differences remain to be elucidated [114–117]. Although it is tempting to suggest that the increased neuronal growth that is observed following damage is beneficial as it is seen following improvements in behavior, it is possible that the growth is promoting compensatory behaviors rather than true recovery [24, 118, 119], which may be interfering with or even preventing true recovery [84, 109, 120–122].

Forced use of the unimpaired limb following stroke, mimicking compensatory use following injury, is associated with decreased neuronal activation [123, 124] and further reduction of forelimb movement representations in the perilesion cortex [84]. Kim and colleagues report not only decreased forelimb representation area in perilesion motor cortex but also an increase in axodendritic synapses and multiple synaptic boutons following forced use of the unimpaired limb (i.e., compensatory limb training) [84]. This synaptic density negatively correlated with functional outcome of the impaired limb, suggesting that aberrant synaptogenesis, potentially of transcallosal projections, may contribute to the poor functional outcome associated with compensatory limb use following injury. Interestingly, animals that have had callosal transections do not exhibit the compensatory limb effect, with forced use of the unimpaired limb having no impact on bad limb recovery [122].

Another way experimental researchers define recovery is by whether the means (i.e., methods) to achieve a particular end point following injury is similar to how it would be performed in the intact animal [104]. Following injury, there is an emergence of what has been regarded as “self-taught” behaviors that develop spontaneously as behavioral deficits begin to subside. These behaviors may develop as a response to compensate for those behaviors lost as a result of injury [125, 126]. For instance, following an ischemic injury to the motor cortex, squirrel monkeys are unable to use their affected hand in reaching for a pellet of food [64]. Over time, there is a gradual return in the ability to use the limb, and this improvement corresponds to reorganization of the motor map [21, 72, 77, 127]. However, recovery of limb movement is due to the use of compensatory behavioral strategies that are fundamentally different from preinjury strategies [128–130]. Even more careful analysis has demonstrated that although the ability to grasp has returned, there are residual fine motor deficits that lead to the development of compensatory movement of individual digits despite further training [73, 131, 132].

In a similar vein, injury to the motor cortex in rats results in an inability to successfully reach for a food pellet that abates over time. Rats with motor cortex lesions are unable to make rotational movements and demonstrate impaired digit use [24]. A more detailed analysis of the reaching behavior in rodents also suggests that even though lesioned animals may regain use of the impaired limb, many qualitative aspects of the behavior are different [133]. Further, a return of the reaching ability in the rat occurs in distinct stages [134].

In the acute stage following ischemic injury, lesioned animals are unsuccessful at their reaching attempts. Erickson et al. [135] suggested that animals are in a sense “learning” that they are likely to be unsuccessful in reaching for the pellet as there is a decline in the number of reaching attempts during this period. This “learned nonuse” occurs only in the acute stages following damage; 8 days post damage, rats increase their number of reach attempts [135]. As animals show an increase in their number of reach attempts, there is a corresponding increase in the number of individually repeated gestures. An animal may advance its limb and withdraw several times without ever grasping the food. Even though there is an increase in the number of attempts, if there is a reduction in the number of successful reaches due to the additional gestures, this behavior could be characterized as “learned bad-use” [136, 137]. Encouraging the unimpaired limb to be used can interfere with later training of the impaired limb [109, 111, 120, 123, 138]. It is possible that remaining motor systems take advantage of the beneficial growth that occurs following ischemic injury, which leads to increased use of the unimpaired limb. Interestingly, bilateral limb use (via either focused, skilled training or home-cage enrichment that encourages dexterous use of both limbs) ameliorates this effect, resulting in a restoration of rehabilitative potential of the impaired limb [109, 123].

It may also be possible for behavioral substitution to take place following injury-induced loss of behavior. Rauschecker [139] found that cats that were deprived of vision early in life can solve a visual maze using tactile sensation that is complimented by expanded cortical representation of their facial vibrissae. Therefore, in the absence of one means for solving a task, it appears that another can substitute and allow for the goal to be accomplished via sensory substitution or a different behavioral strategy. Whishaw et al. [140] demonstrated that hemidecorticate rats are able to learn to successfully navigate a maze, which may suggest that different strategies, possibly mediated by subcortical areas, can be substituted in order for them to successfully navigate. This result is not to be unexpected considering rats are able to substitute one spatial strategy in the absence of another in order to successfully navigate an environment [141].

Part of the difficulty of determining whether true recovery has occurred in the experimental setting has been in the analysis of the behavior. Some authors have even stated that using just a few (often only one) behavioral measures may lead to biased estimates of behavior and that a better assessment of behavior comes from using a “battery” of species-typical and learned behaviors [105]. The use of simple “end point” measures also limits the interpretation of whether a motor behavior has returned [134, 136, 142]. Further, the fractionation of more complex behaviors (e.g., reaching) enables researchers to determine what is possible with regard to recovery and whether animals are using compensatory behaviors [134, 143].

As mentioned previously, most rehabilitative treatments encourage the use of compensatory strategies following injury. However, there are clinical studies of rehabilitative therapies for motor deficits that have focused on training the impaired limb following stroke [144–147]. Some researchers have suggested that what is observed during natural recovery is the development of compensatory behaviors and that “true” recovery is possible following specific rehabilitative training focused on the impairment rather than behavioral outcome [148, 149]. Still, other researchers have taken an extreme stance on the issue suggesting that when recovery is observed, it is due to the reorganization of remaining areas that lead to the development of compensatory behaviors [24]. Even if the behavior is similar to preinjury conditions, the argument is that the remaining functional areas now have to compensate for the loss. Therefore, this argument posits that in order for true recovery to occur, the neurons and their corresponding neural connections that were lost during injury need to be replaced rather than substituted [24, 150].

## 4. The Role of Reserve after Brain Injury

In the field of brain injury, there remains a clear disconnect between brain trauma severity and clinical emergence [151, 152]. A similar brain injury among two, separate individuals may not result in the same degree of behavioral impairments. It has been proposed that this observation could be a result of individual differences in a concept known as cognitive reserve. Cognitive reserve (CR) is considered to be an accumulation of complex neural networks that allows for unique task processing in the brain [153]. CR can be increased through a variety of mental activities that keep the brain active. In other words, “exercising” or strengthening the brain's neuronal connections leads to a large CR. This “exercise” works to provide the brain with a more plastic and varied neural circuitry. CR has been associated with characteristics such as a balanced diet, occupational complexity [154], IQ [152], and participation in various lifestyle activities [155]. According to Murray et al. [156], level of education can also be used as a marker for cognitive reserve. As such, studies have shown that those with high levels of educational attainment have been associated with greater short- and long-term functional recovery after brain injury, in cases of both ischemic stroke [157] and moderate/severe traumatic brain injuries [158, 159].

Another hypothesis that has been put forth is that cognitive reserve is based upon one's entire lifetime. If this is the case, then older individuals should, in theory, have more cognitive reserve capacity than younger individuals since they have had longer to fully develop their more elaborate and intricate communicative system. Although, it must be pointed out that the brain processing in those with high CR is the same for all individuals [153]. The capacity of CR may vary, but its way of action does not change. A larger CR would give an individual a higher threshold for injury and would require an injury of greater severity for clinical symptoms to show [152]. However, research has shown that CR positively influences functional recovery not only in adults but also in children and adolescents [158]. It would seem that a larger CR, at any age or time point during development, could help the brain to better sustain injuries [160]. Although, the literature also suggests that a CR of lower capacity can exacerbate the secondary effects of brain injuries, particularly in instances of TBIs [158]. Therefore, CR may serve as a preventative compensatory mechanism in order to keep the injury from producing further and unnecessary damage after brain injury.

There is the possibility that cognitive reserve may be acting as the nervous system's natural means of compensation after brain injury. Specifically, a large cognitive reserve could provide a more convenient outlet for plasticity to occur, allowing for the more efficient and established neural connections to help the brain better sustain injuries because of its higher level of threshold [159, 161]. In addition to compensatory action through an emphasis on well-established connections, individuals that accumulate a strong CR throughout their lifetime may have a brain that is unique in neural organization in another way, such that the brain communicates in a more holistic manner through a more varied neural network rather than through a limited number of possible neuronal pathways [154]. Since the level of redundancy is high in those with a large CR, it is unlikely that a brain injury will disrupt all communicative routes, thereby fostering a quicker, less intrusive cognitive recovery through the utilization of alternative neural circuitry [162]. It remains to be shown if cognitive reserve is a form of preventive compensation (i.e., the brain is compensating for natural loss because there is synaptic loss as we age and neurons compensate for the loss by increasing synaptic number) [163]. But if there is an injury, this compensation from natural aging gets used up.

## 5. The Impact of Timing and Intensity on Rehabilitation Efficacy

Following CNS injury, experience-induced plasticity (e.g., changes induced via rehabilitation) interacts with the natural richly plastic environment described above. The ultimate efficacy of rehabilitative training depends on how well coordinated those plastic events are. There are a number of factors that may drive more functionally beneficial effects of rehabilitative training, including the timing and intensity of training.

As we have discussed, the injured brain can both promote and inhibit neural plasticity. In order to maximize the functional benefit of rehabilitative training, the timing of training onset must coincide with a naturally more growth permissive environment following injury. Identifying the ideal “window of recovery” following injury is difficult, though research in rodents suggests that there is an early vulnerable period following injury during which training can have negative effects on recovery and exacerbate neural damage. For instance, forced use of the impaired forelimb during the first seven days following stroke results in poor functional outcome and larger lesion size compared to rats permitted to engage either forelimb [164–166]. Similar results are found with exercise as a rehabilitative strategy, with early exercise reducing neuroplasticity-related molecules in the hippocampus following traumatic brain injury in rats. More favorable outcomes are reported if the exercise is initiated two weeks following TBI, with the same molecules being upregulated and spatial memory improving [167, 168]. Early exercise can also be problematic following ischemic stroke causing increased apoptosis and impaired learning performance [169, 170].

When is the best time to introduce rehabilitative training—the question is somewhat difficult. Biernaskie and colleagues found that rehabilitative training was more effective in improving behavioral outcome when initiated five days rather than 30 days following stroke [171]. Similar results were reported by Norrie et al. [172], who found that though stepping function in rats improved after motor rehabilitative training initiated both immediately after spinal cord injury and after a three-month delay, the immediate rehabilitation was much more efficacious. Early onset training is also more beneficial to structural plasticity; motor maps in monkeys exhibit decreased sparing of movement representation areas in the motor cortex when training is initiated one month after ischemic cortical infarct rather than one week [173]. Together, these findings suggest that the ideal time to introduce symptom-specific, skilled rehabilitative training is early, but not immediately, after insult. However, the exact time window for beneficial structural and functional outcomes is still unclear. It is important that we continue to explore the regenerative and degenerative responses of the brain to injury and how these responses interact with behaviorally induced experience-dependent plasticity to drive functional outcome.

Another factor that affects behavioral outcome following injury is the intensity of rehabilitative training. In the intact brain, training intensity impacts both the rate of behavioral change as well as the neural consequences associated with new learning. For instance, mice that receive twice daily motor skill training sessions exhibit a faster acquisition of the skill [174]. MacLellan and colleagues (2011) found that enriched rehabilitation (skilled reaching combined with enriched housing) was only effective in improving functional recovery when enrichment was provided during the more active dark cycle, when rats are more likely to engage with enrichment options at higher intensity [175]. The researchers suggest that there may be a threshold of rehabilitation that is necessary to provide functional benefits. Similarly, Bell et al. [108] found that twice-daily training on the Pasta Matrix Reaching Task (i.e., skilled rehabilitative training) was more beneficial for functional outcome than once-daily training sessions. Specifically, high-intensity training resulted in a faster return to preoperative performance levels [108]. Results from these studies suggest that high-intensity rehabilitative training, initiated early after insult, would be the most effective strategy to employ in humans. It should be noted that current rehabilitative regimens in humans are considerably less intense than those practiced in animal models [22].

## 6. Conclusions

Although the proceeding discussion has focused on compensation/recovery following injury, it is likely that these mechanisms reflect a natural phenomenon that allows an organism to constantly adapt to an ever-changing environment rather than exclusively occurring following injury. While this is a novel consideration in understanding compensation, it has its basis in the observation of what occurs during learning and may reflect the nervous system's ability to adapt to an ever-changing environment (i.e., experience-dependent plasticity). In most normal situations for solving a task, there is a hierarchy of behaviors in order to successfully complete it. If, for instance, an individual is blindfolded, they may still be able to complete a maze through the use of tactile or auditory cues [98]. Also, if a “normal” individual is blindfolded and taught to tacitly discriminate for a prolonged period, there is a temporary increase in activity present in the occipital cortex when tactile stimulation is given [176].

The plastic changes that occur following injury are strikingly similar to those that are observed in normal brains following learning or other experiences [105, 177]. For instance, training of a particular motor sequence induces an altered representation in a normally functioning nervous system that is similar to what occurs following damage [178, 179]. One explanation for this may be that the nervous system is able to change in response to behavioral demand. In many instances, plastic changes occur in at all levels of organization from gene expression to neural systems following injury or during “normal” training [180]. This may suggest that there is a common thread between what occurs following damage and what occurs in situations where plasticity normally occurs. Therefore, one possible method to understand the changes associated with recovery/compensation is observing situations where plasticity normally occurs. However, future work needs to focus on determining and defining what entails complete recovery and whether it is possible for it to occur naturally or through the use of therapeutic intervention or if compensation is the only possibility.
